# Inactivation of class II PI3K-C2α induces leptin resistance, age-dependent insulin resistance and obesity in male mice

**DOI:** 10.1007/s00125-016-3963-y

**Published:** 2016-04-30

**Authors:** Samira Alliouachene, Benoit Bilanges, Claire Chaussade, Wayne Pearce, Lazaros C. Foukas, Cheryl L. Scudamore, Larissa S. Moniz, Bart Vanhaesebroeck

**Affiliations:** UCL Cancer Institute, University College London, 72 Huntley Street, London, WC1E 6DD UK; Galderma R&D, Sophia Antipolis Cedex, France; Institute of Healthy Ageing and Department of Genetics, Evolution and Environment, University College London, London, UK; Mary Lyon Centre, MRC Harwell, Harwell Science and Innovation Campus, Harwell, UK

**Keywords:** Food intake, Glucose homeostasis, Insulin, Insulin resistance, Knock-in leptin, Leptin resistance, Mouse gene targeting, Obesity, PI3K

## Abstract

**Aims/hypothesis:**

While the class I phosphoinositide 3-kinases (PI3Ks) are well-documented positive regulators of metabolism, the involvement of class II PI3K isoforms (PI3K-C2α, -C2β and -C2γ) in metabolic regulation is just emerging. Organismal inactivation of PI3K-C2β increases insulin signalling and sensitivity, whereas PI3K-C2γ inactivation has a negative metabolic impact. In contrast, the role of PI3K-C2α in organismal metabolism remains unexplored. In this study, we investigated whether kinase inactivation of PI3K-C2α affects glucose metabolism in mice.

**Methods:**

We have generated and characterised a mouse line with a constitutive inactivating knock-in (KI) mutation in the kinase domain of the gene encoding PI3K-C2α (*Pik3c2a*).

**Results:**

While homozygosity for kinase-dead PI3K-C2α was embryonic lethal, heterozygous PI3K-C2α KI mice were viable and fertile, with no significant histopathological findings. However, male heterozygous mice showed early onset leptin resistance, with a defect in leptin signalling in the hypothalamus, correlating with a mild, age-dependent obesity, insulin resistance and glucose intolerance. Insulin signalling was unaffected in insulin target tissues of PI3K-C2α KI mice, in contrast to previous reports in which downregulation of PI3K-C2α in cell lines was shown to dampen insulin signalling. Interestingly, no metabolic phenotypes were detected in female PI3K-C2α KI mice at any age.

**Conclusions/interpretation:**

Our data uncover a sex-dependent role for PI3K-C2α in the modulation of hypothalamic leptin action and systemic glucose homeostasis.

**Access to research materials:**

All reagents are available upon request.

**Electronic supplementary material:**

The online version of this article (doi:10.1007/s00125-016-3963-y) contains peer-reviewed but unedited supplementary material, which is available to authorised users.

## Introduction

Phosphoinositide 3-kinase (PI3K)s are a family of lipid kinases that are activated by growth factors, hormones and cytokines, and which control cell growth, proliferation and metabolism. Mammals have eight isoforms of PI3K, divided in three classes [1]. The class I PI3K isoforms (p110α, p110β, p110γ, p110δ) are activated by tyrosine kinases or G protein-coupled receptors (GPCRs) and generate the phosphatidylinositol (3,4,5)-trisphosphate lipid (also known as PIP_3_) important for activating downstream effectors such as Akt/protein kinase B. Among the class I PI3Ks, p110α has been identified as the most important isoform in systemic and hepatic insulin signalling [2–4].

In contrast to the class I PI3Ks, the class II (PI3K-C2α, -C2β and -C2γ) and III (vps34) PI3K isoforms are more enigmatic. Class II PI3Ks have been reported to be activated by a wide range of agonists, such as growth factors, GPCRs and adhesion molecules (reviewed in refs. [1, 5–7]). However, the molecular details of how class II PI3Ks couple to this multitude of upstream receptors remain unclear. Class II PI3Ks are thought to mainly convert the PI lipid to PI(3)P that is involved in vesicular trafficking processes, such as endocytosis and autophagy. PI(3)P effectors include proteins containing FYVE or PX lipid-binding domains. Importantly, class II PI3Ks can also convert PI(4)P to PI(3,4)P_2_ [7–10], which, like PIP_3_, coordinates the localisation and the function of effector proteins containing a PH domain.

Previous cell-based studies have revealed non-redundant functions for the class II PI3K isoforms in a broad variety of biological processes. For instance, PI3K-C2α has been implicated in the regulation of GLUT4 translocation and glucose transport, neurosecretory granule release, insulin secretion, endocytosis and muscle cell contraction, whereas roles for PI3K-C2β in cell migration and K^+^ channel activation have been reported [5, 6, 11].

Knockout (KO) mice for each class II PI3K isoform have been described. KO studies of the gene encoding PI3K-C2α (*Pik3c2a*) have implicated this isoform in angiogenesis [12], generation of the primary cilium [13] and protection against kidney cyst formation [14]. Mice homozygous for a gene-trap *Pik3c2a* allele resulting in strongly reduced expression of a carboxy-terminally truncated PI3K-C2α protein, are viable but develop chronic renal failure [15]. A PI3K-C2β KO mouse line has been created but no phenotypes have been reported in these mice to date [16]. More recently, a study using ΚΟ of PI3K-C2γ, which is mainly expressed in the liver, has provided evidence that this PI3K isoform is a Rab5 effector that positively controls insulin signalling in the liver [10].

In class I PI3K KO mice, remarkable compensation mechanisms by the non-targeted isoforms have been reported, with some class I PI3K KO mice even showing enhanced PI3K signalling (reviewed in [17]). Such phenomena have not been observed in class I PI3K mice in which the endogenous PI3K are inactivated by the introduction of a point mutation in the kinase domain, so called kinase-dead knock-in (KI) mice (reviewed in [17]). The KI strategy also better mimics the impact of systemically administered small molecule inhibitors of PI3K isoforms.

We recently generated PI3K-C2β kinase-dead KI mice and showed that this class II PI3K isoform plays a negative role in insulin signalling and glucose homeostasis [18]. Indeed, PI3K-C2β ΚΙ mice display enhanced insulin sensitivity and glucose tolerance, with enhanced insulin-mediated Akt phosphorylation [18]. Interestingly, PI3K-C2γ KO mice showed the inverse phenotype, displaying insulin resistance and glucose intolerance [10].

Given the roles of PI3K-C2β and PI3K-C2γ in glucose metabolism, and the previous evidence from cell line-based studies for a role for PI3K-C2α in insulin signalling [19–22], we decided to examine the impact of in vivo PI3K-C2α inactivation on glucose homeostasis. This was done in heterozygous PI3K-C2α KI mice, which were viable and fertile, as homozygous inactivation of PI3K-C2α led to embryonic lethality. Unlike in cell lines, where downregulation of PI3K-C2α has been shown to dampen insulin signalling, no changes in organismal insulin sensitivity were observed in PI3K-C2α KI young mice. However, we found that male PI3K-C2α KI mice displayed hypothalamic leptin resistance, leading to age-dependent obesity, insulin resistance and glucose intolerance.

## Methods

### Mice

Mouse gene targeting was performed by Artemis (Cologne, Germany) in C57BL/6NT embryonic stem cells. Mice were backcrossed on the C57BL/6J strain (Charles River, Margate, UK) for three to five generations. *Pik3c2α*^tm1521(D1268A)Arte^ mice used for experiments were on a mixed C57BL/6J × C57BL/6NT background, with wild-type (WT) littermates used as controls. Experiments involving mice were approved by the local ethics committee.

The experimenters were not blind to group assignment and outcome assessment.

The primers used for genotyping are: forward primer: 1470-29 KI: GACTGATTGGGATACAAACCC, antisense primer: 1470-30 KI: GCTCTGAGCTGCAGATATGG with expected fragments: 320 bp (WT), 452 bp (KI), 320 bp (WT) + 452 bp (KI) under the following PCR conditions: 95°C for 5 min, 34 times (95°C for 30 s, 60°C for 30 s, 72°C for 1 min) and 72°C for 10 min.

### Lipid kinase assays in vitro

PI3K activity assays using PtdIns as a lipid substrate were performed as described [23].

### Western blot analysis and antibodies

See electronic supplementary material (ESM) Methods for further details.

### In vivo insulin signalling and other metabolic analyses

were performed as described [18]. Serum levels of leptin were measured using ELISA kit (Millipore, Billerica, MA, USA). The pyruvate tolerance test (PTT) was performed by i.p. injection of pyruvate (Sigma-Aldrich, St Louis, MO, USA; 2 g/kg) after an 18 h fast.

### Food intake and leptin sensitivity analysis

Mice were singly housed and allowed to acclimatise for a week before the study. Food intake was measured daily and results expressed as cumulative food intake (g of chow). Mice were injected daily with vehicle (55% 15 mmol/l HCl/45% 7.5 mmol/l NaOH, i.p. vol/vol) for 3 days. After 1 week of recovery, the same mice were injected daily with leptin (from R&D systems, Minneapolis, USA; 2.5 mg/kg, i.p.) for 3 days, 2 h before the onset of the dark cycle. For analysis of leptin signalling in the hypothalamus, leptin (2 mg/kg) or vehicle were injected i.p., followed 30 min later by harvesting and freezing of the hypothalamus in liquid nitrogen. Experiments were performed on 12-week-old mice.

### Histology

For tissue sections, haematoxylin and eosin (HE) staining was performed on 5 μm paraffin sections of tissues fixed for 16 h in 4% paraformaldehyde (PFA) in PBS at 4°C. For staining of neutral lipids, cryosections were fixed and stained with Oil Red O (Sigma-Aldrich). In brief, sections were fixed with 4% PFA in PBS at room temperature for 15 min. Fixed sections were washed again with PBS and stained with Oil Red O (0.5% wt/vol. isopropanol, diluted 3:2 in PBS) for 1 h at room temperature. Stained sections were rinsed in 60% isopropanol, followed by deionised water and mounted in Vectashield. To randomise the condition of tissue collection, two cohorts of mice were used for histology of metabolic tissues. The first set of animals was randomly fed and perfused with PFA (4%) before collection of the relevant organs. The second set of mice was starved for 4 h before tissue sampling.

### Determination of adipocyte size

To determine the size of (white) adipocytes, at least 150 adipocytes from representative sections per fat pad (epididymal and perirenal white adipose tissue [WAT]) per mouse (four per genotype) were performed on a Macintosh computer using the public domain NIH Image J program (developed at the U.S. National Institutes of Health and available at http://rsb.info.nih.gov/nih-image/) and the mean value designated as an index of cell size.

### HOMA-IR

was calculated using glucose and insulin concentrations obtained after an 16 h fast, using the following formula: [fasting glucose (mmol/l) × fasting insulin (pmol/l)]/135.

### Statistical analysis

All data are shown as mean values ± SEM, unless otherwise indicated. Datasets were compared for statistical significance using the two-tailed Student’s *t* test or ANOVA where appropriate. Statistical significance is indicated as follows: *p < 0.05; **p < 0.01; ***p < 0.001. The number of animals in each group is indicated by *n*. No results were omitted or excluded from our study.

## Results

### Generation of PI3K-C2α kinase-dead KI mice

**Fig. 1:**
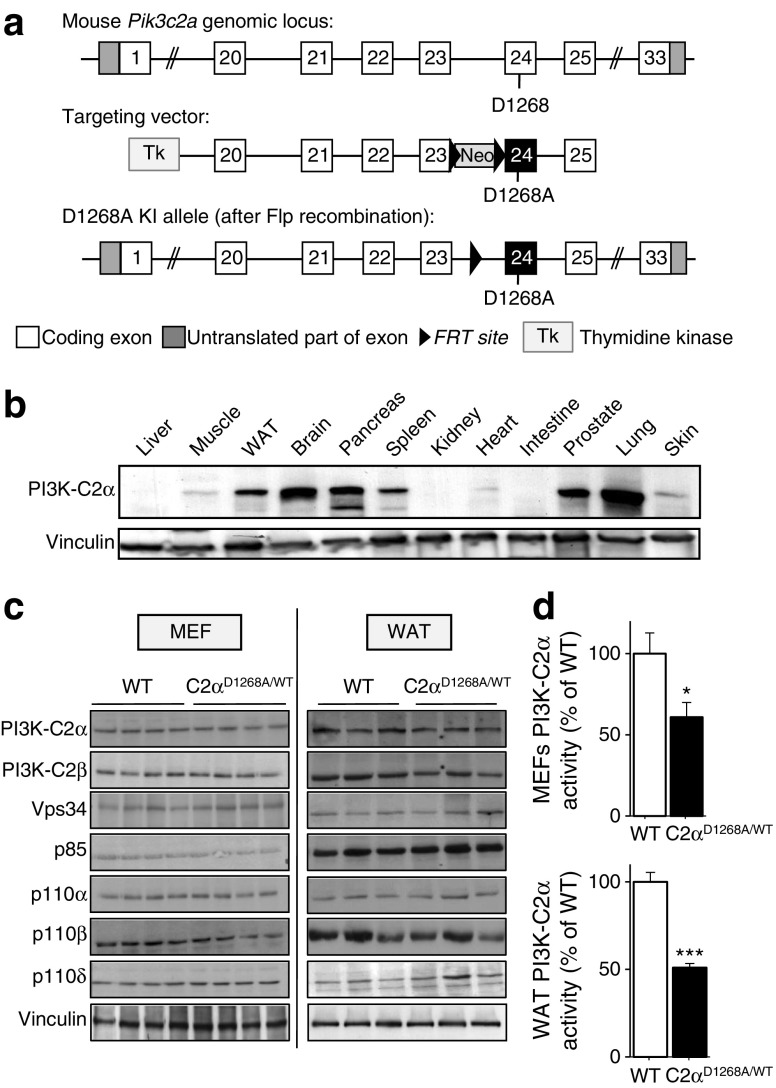
Generation and characterisation of C2α^D1268A/WT^ KI mice. (**a**) Gene targeting strategy to introduce the D1268A mutation in the DFG motif in exon 24 of the *Pik3c2a* gene. The FRT-flanked cassette encoding the *Pgk Neo* selection marker was removed in vivo by breeding onto ACTB-Flp mice. (**b**) PI3K-C2α protein expression. Tissue homogenates were analysed by SDS–PAGE and immunoblotting using anti-PI3K-C2α antibody. (**c**) PI3K isoform expression in WT and C2α^D1268A/WT^ cells and tissue. Each lane on the SDS–PAGE gel represents an independent mouse. Homogenates of MEFs or epididymal WAT from male mice were analysed by SDS–PAGE and immunoblotting using the indicated antibodies. (**d**) Lipid kinase activity associated with PI3K-C2α in WT and C2α^D1268A/WT^ mice. Homogenates of MEFs or epididymal WAT from male mice were immunoprecipitated using PI3K-C2α antibody, and subjected to an in vitro PI3K activity assay. Results shown are pooled data from three independent experiments (each with 2–4 experimental replicates). *p < 0.05; ***p < 0.001

We created a germline KI mouse line in which the genomic DNA encoding the ATP-binding DFG motif in the gene encoding PI3K-C2α (*Pik3c2a*) is mutated to encode the AFG sequence, resulting in the production of a kinase-dead PI3K-C2α protein, further referred to as C2α^D1268A^ (Fig. 1a). Mice heterozygous for the mutated *Pik3c2a* allele (hereafter called C2α^D1268A/WT^ mice; WT indicates the wild-type *Pik3c2a* allele) were born at the expected Mendelian ratios, whereas homozygous C2α^D1268A/D1268A^ embryos could not be recovered beyond embryonic day 10.5–11.5. This observation is consistent with the reported lethality of homozygous PI3K-C2α KO embryos around the same time of development, as a consequence of impaired vascular angiogenesis [12] and impaired hedgehog signalling from defective primary cilia [13]. At present, it is unclear whether the underlying molecular mechanism of lethality in the C2α^D1268A/D1268A^ embryos differs from the PI3K-C2α KO model.

The PI3K-C2α protein showed a broad tissue distribution with high expression levels in some tissues, including muscle, WAT, brain, pancreas, spleen, prostate and lung (Fig. 1b). In line with previous observations using the KI gene targeting strategy, expression of the kinase-dead PI3K-C2α protein, and that of other PI3K isoforms, was not significantly altered either in mouse embryonic fibroblasts (MEFs) derived from E13.5 embryos or in WAT from adult mice (Fig. 1c). PI3K-C2α immunoprecipitates from tissues and C2α^D1268A/WT^ MEFs displayed a ~50% reduction in associated in vitro lipid kinase activity (Fig. 1d), consistent with heterozygous inactivation of PI3K-C2α.

### Normal glucose homeostasis and insulin sensitivity in C2α^D1268A/WT^ young mice

**Fig. 2:**
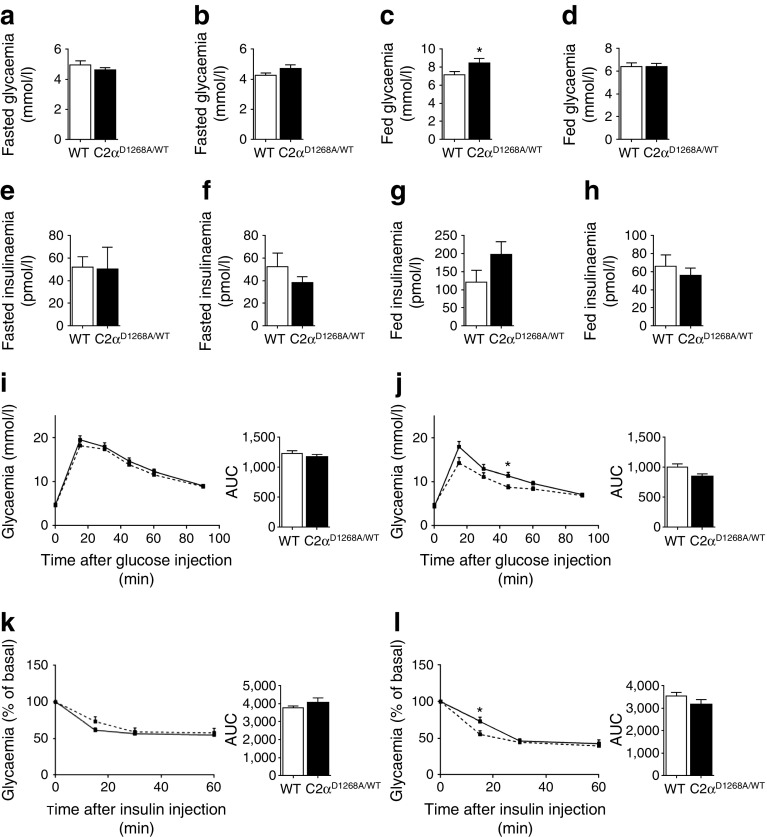
Normal glucose homeostasis and insulin sensitivity in 12-week-old C2α^D1268A/WT^ mice and hyperleptinaemia in 12-week-old male C2α^D1268A/WT^ mice. Data represent mean ± SEM. (**a**–**d**) Fasted glycaemia in males (**a**) and in females (**b**) and fed glycaemia in males (**c**) and in females (**d**). *n* = 20–32 mice were used. (**e**–**h**) Plasma insulin levels under fasted condition in males (**e**) and in females (**f**) and under fed condition in males (**g**) and in females (**h**). *n* = 7–16 mice were used. (**i**–**j**) GTT. Results shown are pooled data from four or five independent experiments for male (**i**) and female (**j**) mice. The AUC is shown. *n* = 12–28 mice were used. (**k**, **l**) ITT. Results shown are pooled data from three independent experiments for male (**k**) and for female (**l**) mice. The AUC is shown. *n* = 8–13 mice were used. Solid line, WT; dashed line, C2α^D1268A/WT^. *p < 0.05

Heterozygous mice were viable and fertile, with no apparent defects as assessed by histopathological analysis of a broad range of tissues of up to 12 month-old mice (see ESM Table 1]. Class II PI3K overexpression and RNA interference (RNAi)-based studies in cell lines previously documented a role for PI3K-C2α in insulin-stimulated glucose uptake [24] and insulin secretion [25, 26]. Therefore, we first monitored metabolic variables in C2α^D1268A/WT^ mice. Levels of blood glucose (Fig. 2a–d) and plasma insulin (Fig. 2e–h) in overnight-fasted or randomly fed states were unaffected in both 12-week-old C2α^D1268A/WT^ male and female mice, although a slight hyperglycaemia and a tendency to hyperinsulinaemia was observed in male mice under fed conditions (Fig. 2c, g). Overall glucose clearance upon injection of glucose (Fig. 2i, j) or insulin (Fig. 2k, l) was not significantly affected in mice of both sexes (no statistically significant differences in the AUCs), although a mild (but statistically non-significant) tendency for improvement of glucose and insulin tolerance was observed in female mice (Fig. 2j, l).

In line with the lack of metabolic defects, phosphorylation of Akt in liver, muscle and fat was similar in 12-week-old WT and C2α^D1268A/WT^ mice upon insulin injection (ESM Fig. 1a and data not shown). These data indicate that organismal glucose homeostasis and insulin signalling are not critically dependent on PI3K-C2α activity.

### Increased leptin levels and leptin resistance in male C2α^D1268A/WT^ mice

**Fig. 3:**
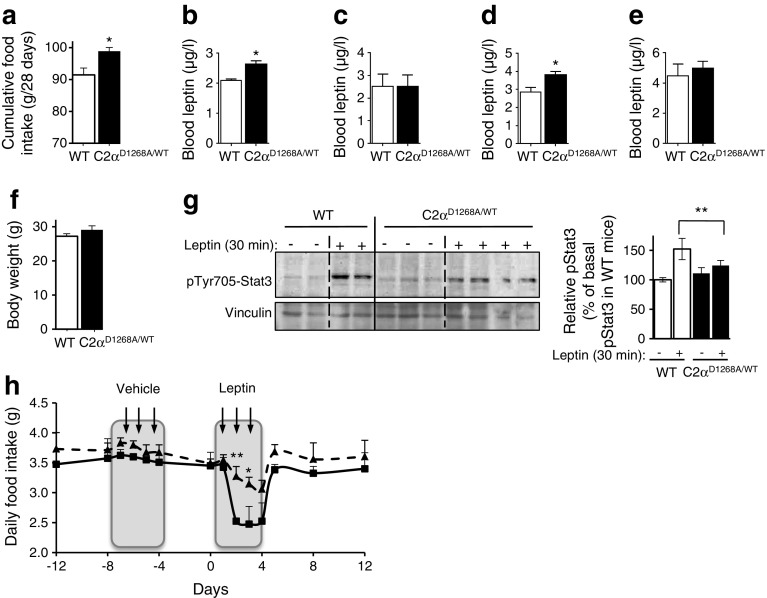
Leptin resistance in male 12-week-old C2α^D1268A/WT^ mice. Data represent mean ± SEM, except for (**f**) mean ± SD. (**a**) Cumulative food intake over a 28-day period in 12-week-old mice. *n* = 4/7 (WT/C2α^D1268A/WT^) mice were used. (**b**–**e**) Serum leptin levels in 12-week-old male (**b**) and female (**c**) mice and in 32-week-old male (**d**) and female (**e**) mice. *n* = 8–13 (WT/C2α^D1268A/WT^) mice were used. (**f**) Whole-body weight of 12-week-old mice. *n* = 6 mice were used. (**g**) Hypothalamic homogenates isolated from 12-week-old mice 30 min after i.p. injection of 2.5 mg/kg leptin were analysed by SDS–PAGE and immunoblotting using the indicated antibodies. Representative western blots are shown and quantification is based on pooled data from three independent experiments with 2–4 mice/condition/experiment. Each lane on the SDS–PAGE gel represents an independent mouse. (**h**) Defective functional response to exogenous leptin in 12-week-old mice. The data show food intake, before and after daily injection of vehicle or leptin. *n* = 5–7 mice were used. Solid line, WT; dashed line, C2α^D1268A/WT^. *p < 0.05; **p < 0.01

Although daily food consumption was not significantly affected, cumulative food intake was enhanced over a period of 7 days (data not shown) and 28 days in male (Fig. 3a) but not in female mice (data not shown). Leptin, an adipose-derived hormone, is known to suppress appetite by interacting with the leptin receptor (LEPRb) in the hypothalamus [27, 28]. Circulating levels of leptin were approximately 25% higher in both young (12 weeks) and old (32 weeks) male C2α^D1268A/WT^ mice compared with WT mice (Fig. 3b, d), with no changes in female C2α^D1268A/WT^ mice (Fig. 3c, e). For this reason, we focused further investigations only on male mice.

In addition to leptin’s well-known sexual dimorphism, with males known to be more sensitive to leptin than females [29, 30], body adiposity plays a crucial role in modulating leptin levels [29]. We found that, in young male C2α^D1268A/WT^ mice, the body weight was unaffected compared with WT mice (Fig. 3f), with no changes in different organ weight (including WAT) apart from a slight reduction of the kidney weight (ESM Fig. 1b–f). In addition, no significant histopathological findings were observed in HE-stained kidneys (data not shown). These data show that the increased leptinaemia does not result from increased adiposity.

As a consequence, the higher levels of circulating leptin most likely arose as a compensatory mechanism for leptin resistance [31]. Indeed, in response to injection of leptin, hypothalami from young (12-week-old) male C2α^D1268A/WT^ mice showed markedly reduced phosphorylation of Stat3, a downstream effector of leptin (Fig. 3g). In line with these observations, systemically administered leptin had a severely reduced ability to acutely reduce food intake in C2α^D1268A/WT^ male mice, compared with WT mice (Fig. 3h). As expected, the hypothalamic response to leptin injection was unaffected in female C2α^D1268A/WT^ mice (ESM Fig. 1g). Taken together, these results demonstrate that hypothalamic leptin signalling is defective in young (12-week-old) male C2α^D1268A/WT^ mice, giving rise to an early onset leptin resistance.

### Age-dependent obesity in male C2α^D1268A/WT^ mice

**Fig. 4:**
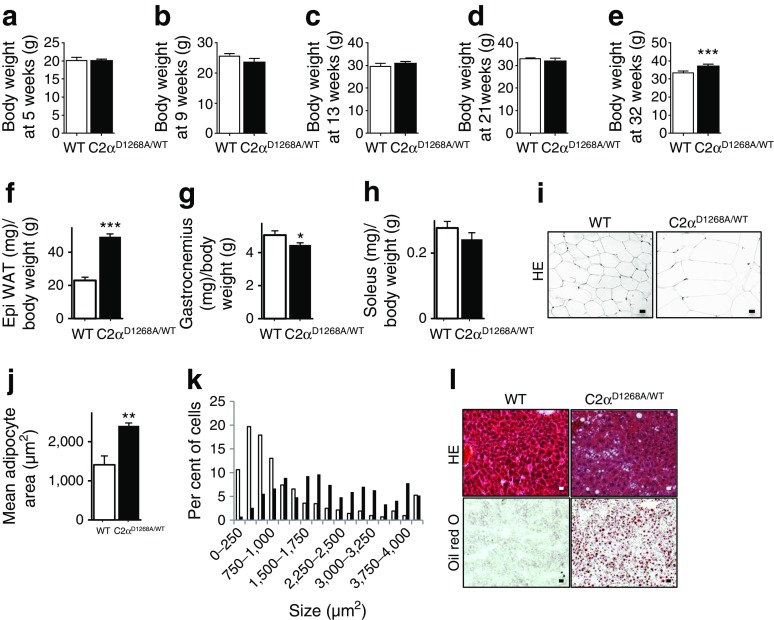
Age-dependent adiposity in male 32-week-old C2α^D1268A/WT^ mice. Data represent mean ± SEM. (**a**–**e**) Whole-body weight variation upon ageing of male mice at 5 weeks old (**a**), 9 weeks old (**b**), 13 weeks old (**c**), 21 weeks old (**d**) and 32 weeks old (**e**). (**f**–**h**) Organ weight to body weight ratios from 32-week-old mice. Epididymal WAT (**f**), gastrocnemius (**g**) and soleus (**h**). *n* = 6–8 mice were used. (**i**–**k**) Epididymal WAT histology from 32-week-old mice. HE staining of epididymal WAT sections is shown (**i**). Mean adipocyte areas (**j**) and adipocyte area distribution profiles (**k**) are also shown. Data are representative of epididymal WAT sections from individual mice. *n* = 9–12 mice were used. Scale bar: 20 μm. White bars, WT; black bars, C2α^D1268A/WT^. (**l**) Liver histology. HE staining and Oil Red O staining of liver sections. Data are representative of liver sections from *n* = 9–12 mice. Scale bar: 20 μm. *p < 0.05; **p < 0.01; ***p < 0.001

Mice and humans with leptin-deficiency (*ob/ob*) or leptin resistance (*db/db*) are obese and hyperphagic [27, 31–35]. Accordingly, and in line with the elevated cumulative food intake, male C2α^D1268A/WT^ mice displayed a mild, adult-onset obesity under normal chow diet. Male C2α^D1268A/WT^ mice showed a similar body weight to WT mice until 21 weeks of age and reach a 12% increase in body weight compared with WT mice at 32 weeks of age (Fig. 4a–e). In line with their normal circulating levels of leptin (Fig. 3c, e), female C2α^D1268A/WT^ mice exhibited a similar weight gain to WT mice (ESM Fig. 1h).

We next investigated the weight gain phenotype in male mice in more detail. Nose-to-tail length was unaffected in both male and female C2α^D1268A/WT^ mice (data not shown), suggesting that the weight gain observed was not due to increased animal growth. Thirty-two-week-old male C2α^D1268A/WT^ mice displayed increased adiposity (Fig. 4f) and loss of relative muscle mass (Fig. 4g, h). Histological analysis, confirmed by consistent sampling and using robust morphometric analysis, revealed enlarged adipose cells in epididymal (Fig. 4i–k) and perirenal WAT (ESM Fig. 1i–k) in 50% of the C2α^D1268A/WT^ mice analysed. Consistent with the development of obesity, more than half of the male C2α^D1268A/WT^ mice had increased lipid accumulation in the liver, as assessed by HE staining (hepatocellular vacuolation), and enhanced Oil Red O staining (Fig. 4l).

Taken together, these data indicate that the leptin resistance observed in male C2α^D1268A/WT^ mice is accompanied by age-dependent obesity, with an increase in adipose tissue and enhanced hepatic lipid accumulation.

### Hyperglycaemia and insulin resistance in obese male C2α^D1268A/WT^ mice

**Fig. 5:**
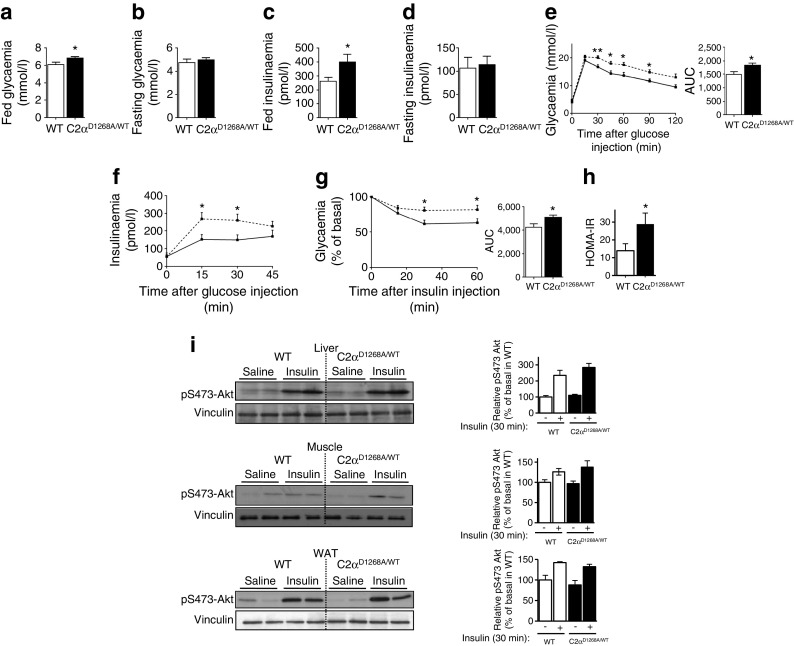
Hyperglycaemia, glucose intolerance and insulin resistance in male 32-week-old C2α^D1268A/WT^ mice. Data represent mean ± SEM. (**a**, **b**) Glycaemia under fed (**a**) and fasted (**b**) conditions of 32-week-old mice. *n* = 16/39 mice were used. (**c**, **d**) Plasma insulin levels under fed (**c**) and fasted (**d**) conditions of 32-week-old mice. *n* = 13/22 mice were used. (**e**) GTT showing glycaemia and (**f**) insulinaemia. Results shown are five independent experiments using mice from different litters. The AUC is shown. *n* = 14–31 (for glycaemia); *n* = 9–11 (for insulinaemia) mice were used. (**g**) ITT. Results shown are pooled data from three independent experiments using independent litters. The AUC is shown. *n* = 9–14 mice were used. (**h**) HOMA-IR index. *n* = 8–11 mice were used. (**i**) Insulin signalling in liver, muscle (gastrocnemius) and WAT of 32-week-old mice. Homogenates from mice injected i.p. with 0.75 U/kg insulin (30 min) were analysed by SDS–PAGE and immunoblotting using the indicated antibodies. Representative western blots are shown and quantification is based on pooled data from two independent experiments with 2–4 mice/condition/experiment. Each lane on the SDS–PAGE gel represents an independent mouse. Solid line, WT; dashed line, C2α^D1268A/WT^. *p < 0.05; **p < 0.01

We next assessed the impact of the late-onset obesity on glucose homeostasis and insulin sensitivity. Under fed, but not under fasted conditions, 32-week-old C2α^D1268A/WT^ males displayed hyperglycaemia (Fig. 5a, b) and hyperinsulinaemia (Fig. 5c, d), with reduced glucose clearance, despite an observed elevated insulin secretion during the glucose tolerance test (GTT) (Fig. 5e, f). This was correlated with insulin resistance, as observed during the insulin tolerance test (ITT) (Fig. 5g). We quantified insulin resistance by calculating the HOMA-IR index. Thirty-two-week-old C2α^D1268A/WT^ male mice showed an increase in the HOMA-IR index (Fig. 5h), which correlated with the impaired insulin sensitivity observed in these mice compared with WT. In line with their normal leptinaemia (Fig. 3c, e) and weight gain (ESM Fig. 1h), female C2α^D1268A/WT^ mice exhibited similar glycaemia, insulinaemia and glucose clearance during GTT or ITT as WT mice (ESM Fig. 2a–f).

Unexpectedly, 32-week-old C2α^D1268A/WT^ and WT mice showed similar levels of insulin-induced Akt phosphorylation in the liver, muscle and adipose tissue (Fig. 5i). Moreover, a PTT (ESM Fig. 2g) displayed no differences between the C2α^D1268A/WT^ and WT mice indicating that gluconeogenesis from pyruvate was unaffected in C2α^D1268A/WT^ mice.

## Discussion

In order to assess the role of the kinase activity of the class II PI3K-C2α, we created a mouse model in which this PI3K isoform has been rendered inactive by introduction of a germline KI mutation in the conserved DFG motif of the ATP-binding site. Recently this mouse model facilitated the uncovering of a new role of PI3K-C2α in platelet membrane morphology [36].

The roles of the class I PI3Ks on metabolism and energy homeostasis have been well-documented [2–4, 23, 37]. Recently, PI3K-C2β and PI3K-C2γ were shown to play a role in glucose metabolism and insulin signalling in vivo [10, 18], with no in vivo metabolic phenotypes of PI3K-C2α reported to date. In the present study, we examined the role of PI3K-C2α in whole-body insulin sensitivity and glucose homeostasis.

Our data reveal that systemic ablation of half of the activity of PI3K-C2α induces leptin resistance in male mice, with a primary defect in leptin signalling in the hypothalamus. Despite normal body weight and adiposity, young C2α^D1268A/WT^ mice had higher circulating leptin levels than WT littermates, and displayed mild hyperphagia. Partial inactivation of PI3K-C2α dampened the reduction in food intake upon exogenous leptin injection, and impaired hypothalamic leptin signalling. In line with this defective leptin responsiveness, partial inactivation of PI3K-C2α led to the development of adult-onset obesity, with accompanying hypertrophy of the adipose tissue, abnormal hepatic lipid accumulation, glucose intolerance, hyperinsulinaemia and insulin resistance. The development of these defects upon ageing was not the result of an intrinsic defect in insulin signalling in insulin-sensitive tissues (WAT, liver and skeletal muscle), as indicated by unaltered insulin-induced phosphorylation of Akt. In addition to the mild hyperphagia observed in C2α^D1268A/WT^ mice, we cannot rule out a possible role of PI3K-C2α inactivation in reducing energy expenditure in the development of the observed age-onset obesity.

### PI3K-C2α in insulin signalling and secretion

Our data show that in vivo, full PI3K-C2α activity is not required for insulin signalling at any stage of adult development. Indeed, young C2α^D1268A/WT^ mice displayed normal insulin signalling in all tissues tested and, as a result, had unaffected systemic insulin sensitivity and glucose tolerance. These observations are in sharp contrast with the previous cell-based studies that reported a positive role for PI3K-C2α in insulin signalling, glucose uptake and insulin secretion [24–26]. The reason for these discrepancies is unclear at the moment. While it is conceivable that data obtained in tissue culture cells do not translate to primary tissues, it is also possible that a more substantial inhibition of PI3K-C2α than heterozygous inactivation is required to interfere with insulin signalling. All KI mice heterozygous for a kinase-dead allele that we have generated thus far, including PI3K-C2α KI mice [36] have been found to display defects in signalling and other phenotypes (p110α: [2, 38]; p110β: [39]; p110δ: [40, 41]). We have also observed metabolic phenotypes in heterozygous PI3K-C2β and vps34 KI mice (our unpublished results). These phenotypes are especially detectable at non-saturating doses of stimulus (including insulin signalling in the case of p110α [2]). We therefore expected the KI gene targeting strategy applied to PI3K-C2α to give rise to partial, stimulus dose-dependent phenotypes, but it is possible that a more substantial (>50%) inhibition of PI3K-C2α might be required to interfere with the biological responses investigated. It is important to mention that the previously reported cell-based studies made use of PI3K-C2α RNAi and overexpression strategies which affect both the kinase activity (as in the current study) as well as potential scaffolding functions of PI3K-C2α [17].

Interestingly, at old age, male C2α^D1268A/WT^ mice did display glucose intolerance and insulin resistance, greatly contrasting the unaltered insulin signalling observed in insulin-sensitive tissues. As mentioned above, it is possible that a more substantial inhibition of PI3K-C2α (>50% inhibition) is required to interfere with insulin signalling.

### PI3K-C2α in leptin signalling

Our data show that PI3K-C2α activity is critical for leptin signalling in the brain. Interestingly, a previous study has presented evidence for leptin-induced activation of PI3K-C2α in macrophages [42]. In addition to actions in the brain, leptin also directly acts on multiple peripheral tissues, including pancreatic islets, liver, adipose tissue, kidney and skeletal muscle [43, 44]. However, genetic deletion of the leptin receptor in these tissues does not alter energy balance, body weight or glucose homeostasis [43, 44], highlighting a more critical role for leptin signalling in the brain with regards to organismal metabolism.

Leptin has also been shown to play an important role in renal pathophysiology [45, 46]. Despite a small decrease in the relative weight of the kidneys in aged C2α^D1268A/WT^ male mice in relation to overall body weight, we did not observe any histopathological abnormalities in the kidneys of male or female heterozygous C2α^D1268A/WT^ mice. However, previously described PI3K-C2α gene-trap mice, which have >75% reduction in organismal PI3K-C2α activity, develop chronic renal failure and a range of kidney lesions, even at a young age [15]. It would be interesting to assess whether a metabolic phenotype is also observed in PI3K-C2α gene-trap mice and whether leptin resistance in kidney cells could contribute to their kidney phenotype.

Finally, the sexual dimorphism of the leptin resistance phenotype in the C2α^D1268A/WT^ mice is in line with the known sexual dimorphism in leptin biology in rodents or humans at the level of serum leptin levels, leptin receptor expression and signalling [29]. Indeed, serum leptin and receptor levels are significantly higher in female mice than in male with equivalent body fat mass [30], which might render females less sensitive to dysfunctions in leptin signalling.

It is most likely that the increase in leptin levels observed in male C2α^D1268A/WT^ mice is a consequence of a hypothalamic leptin resistance, with the adipose tissue (which is not increased in size at young age) secreting more leptin to compensate for the decrease in leptin signalling in the hypothalamus. However, we cannot exclude the possibility that PI3K-C2α could also be involved in leptin secretion from the WAT.

At present, the mechanism of leptin resistance in the male C2α^D1268A/WT^ mice is unclear. Leptin resistance can be due to a multitude of mechanisms, including defective leptin transport from the blood circulation to the brain, defective hypothalamic neural circuitry that regulates energy homeostasis and/or altered leptin signalling [44]. Leptin-induced Stat3 phosphorylation was found to be reduced in the hypothalamus in C2α^D1268A/WT^ mice, indicating that the defect occurs at the level of LEPR signalling. PI(3)P and PI(3,4)P_2_ are lipid products of PI3K-C2α that have been implicated in intracellular vesicular transport, including endocytosis and exocytosis [5, 6]. Importantly, PI3K-C2α has been shown to be concentrated in the trans-Golgi network [47]. It is therefore tempting to speculate that PI3K-C2α is involved in LEPR trafficking. LEPRb, the functional isoform of the leptin receptor, mainly resides in the trans-Golgi network and endosomes, with only a relatively small number of receptors on the plasma membrane to mediate leptin signalling [44]. Interestingly, deletion of Bardet-Biedl syndrome (BBS) proteins, known to promote LEPRb trafficking, impairs leptin signalling and results in leptin resistance and obesity, both in mouse models and in humans [48–50]. In addition to regulating LEPRb trafficking, BBS proteins also play a critical role in the regulation of cilia length as shown in renal medullary cells [51]. In these cells, inactivation of BBS proteins leads to shorter cilia, a phenotype also observed in PI3K-C2α heterozygote KO mice [14]. Cilia are important cellular structures that regulate signalling cascades that affect feeding and satiety [52] but are not directly involved in leptin signalling [53]. Conversely, leptin levels have been shown to regulate neuronal cilia length [52]. Altogether, these data point to a potential role for BBS proteins downstream of PI3K-C2α, impacting both on leptin signalling and cilia length, which could directly and indirectly, respectively, affect organismal metabolism.

In summary, our studies describe the phenotypic characterisation of the first mouse model with a kinase-dead PI3K-C2α and reveal a role for this PI3K in organismal leptin signalling and age-dependent regulation of glucose homeostasis in males.

## Electronic supplementary material

Below is the link to the electronic supplementary material.

ESM Materials and methods (PDF 251 kb)

ESM Fig. 1 (PDF 532 kb)

ESM Fig. 2 (PDF 267 kb)

ESM Table 1 (PDF 221 kb)

## Abbreviations

BBS: Bardet-Biedl syndrome
GPCR: G protein-coupled receptor
GTT: Glucose tolerance test
HE: Haematoxylin and eosin
ITT: Insulin tolerance test
KI: Knock-in
KO: Knockout
LEPR: Leptin receptor
MEF: Mouse embryonic fibroblast
PFA: Paraformaldehyde
PI3K: Phosphoinositide 3-kinase
PIP_3_: Phosphatidylinositol(3,4,5)-trisphosphate
PTT: Pyruvate tolerance test
RNAi: RNA interference
WAT: White adipose tissue
WT: Wild-type
