# Supplementary material for: Inactivation of class II PI3K-C2α induces leptin resistance, age-dependent insulin resistance and obesity in male mice
Source: Diabetologia. 2016 Apr 30;59:1503–12. doi: 10.1007/s00125-016-3963-y (PMC4901096; doi:10.1007/s00125-016-3963-y)
Supplement: Supplementary file 1 — (PDF 251 kb) [file 125_2016_3963_MOESM1_ESM.pdf]

Inactivation of class II PI3K-C2α induces leptin resistance, age-dependent insulin resistance and obesity in male mice

Electronic Supplementary Methods

Western blot analysis and antibodies Proteins were extracted from tissues in lysis buffer containing 137 mM NaCl, 20 mM Tris.HCl pH7.4, 10% (vol/vol) glycerol, 1% (vol/vol) Nonidet P-40 (NP40), 10 mM EDTA, 1 mM EGTA, 1 mM β-glycerophosphate, 20 µM leupeptin, 18 µM pepstatin, 1 mM AEBSF, 4 µg/ml aprotinin, 2 mM Na_3VO_4, 20 mM NaF and 1 mM DTT). The homogenate was cleared by centrifugation at 4°C for 20 min at 15,000g and the supernatant fraction recovered. Protein concentration was determined by colorimetric assay (BCA, Pierce). Homogenates were resolved by SDS-PAGE, transferred to nitrocellulose membranes and probed with specific antibodies overnight at 4°C. Antigen-specific binding of antibodies was visualized by ECL. Western blotting was performed using antibodies to PI3K-C2α p170 (1:1000) from BD Biosciences (#611046); PI3K-C2β (1:1000) from BD Biosciences (#611342); p85 antibody was used as described previously [1]; p110β (1:500) and p110δ (1:500) from Santa Cruz (SC #602 and #7176, respectively); vinculin (1:5000) from Sigma (#V9131), p110α (1:1000), Vps34 (1:1000), phosphoTyr705 Stat3 (1:1000), phosphoS473 Akt (1:1000) from Cell Signaling Technology (CST #4249, #3811, #9145, #9271 respectively). Immunoprecipitation was performed using a homemade PI3K-C2α p170 antibody (SK193, rabbit antibody).

Reference

[1]. Shepherd PR, Nave BT, Rincon J, et al. (1997) Differential regulation of phosphoinositide 3-kinase adapter subunit variants by insulin in human skeletal muscle. J Biol Chem 272: 19000-19007
