# Supplementary material for: Inactivation of class II PI3K-C2α induces leptin resistance, age-dependent insulin resistance and obesity in male mice
Source: Diabetologia. 2016 Apr 30;59:1503–12. doi: 10.1007/s00125-016-3963-y (PMC4901096; doi:10.1007/s00125-016-3963-y)
Supplement: Supplementary file 2 — (PDF 532 kb) [file 125_2016_3963_MOESM2_ESM.pdf]

ESM Fig.1

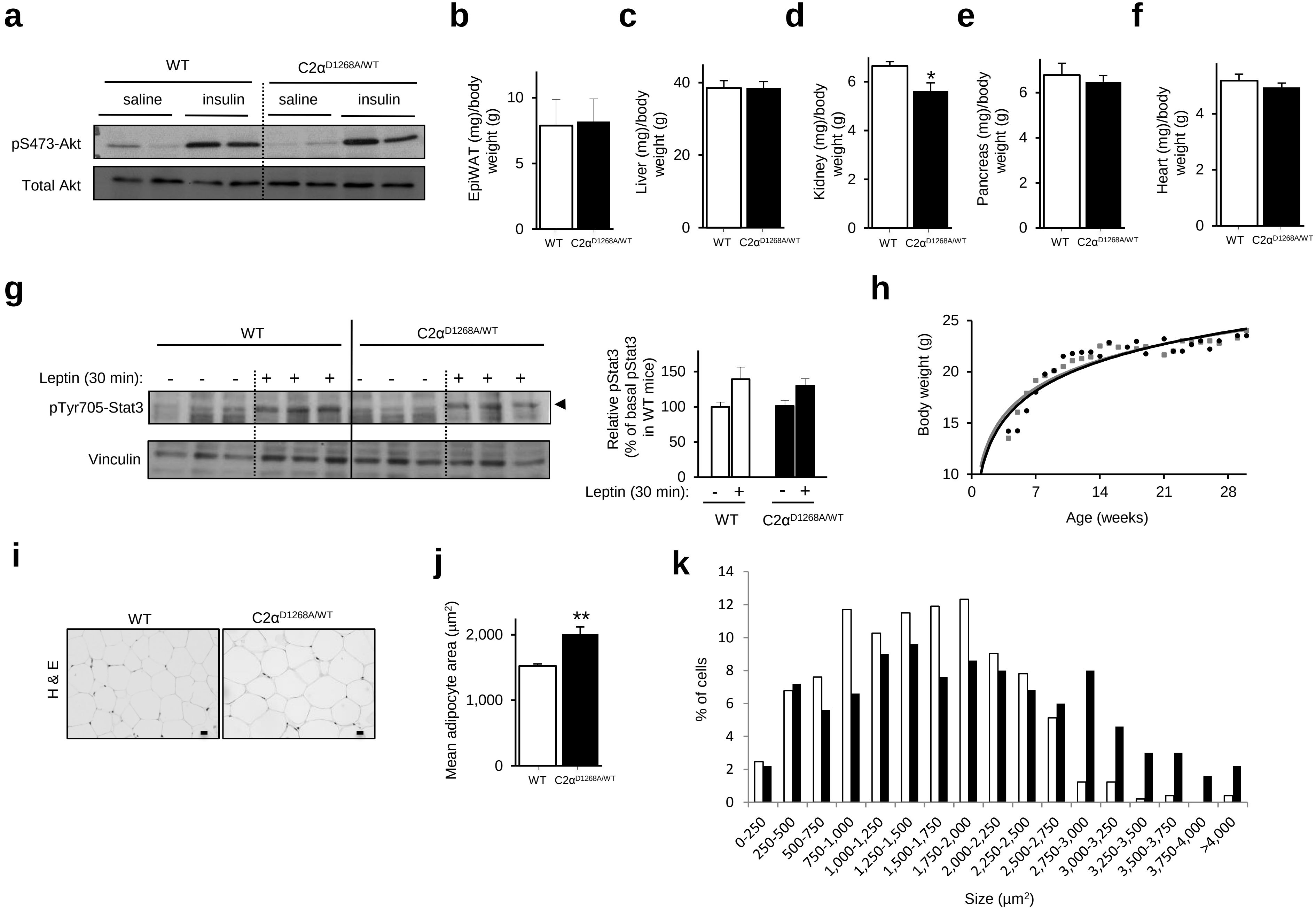

a
WT
C2α D1268A/WT
saline
insulin
saline
insulin
pS473-Akt
Total Akt
b
EpiWAT (mg)/body weight (g)
WT
C2α D1268A/WT
c
Liver (mg)/body weight (g)
d
Kidney (mg)/body weight (g)
*
e
Pancreas (mg)/body weight (g)
f
Heart (mg)/body weight (g)
g
Leptin (30 min):
pTyr705-Stat3
Vinculin
Relative pStat3 (% of basal pStat3 in WT mice)
h
Body weight (g)
Age (weeks)
i
H & E
j
Mean adipocyte area (µm2)
**
k
% of cells
Size (µm2)

Inactivation of class II PI3K-C2α induces leptin resistance, age-dependent insulin resistance and obesity in male mice

Electronic Supplementary Material

ESM Fig.1 Metabolic characteristics and signalling in WT and C2α$^{D1268A/WT}$ mice. Data represent mean ± SEM.
(a) Insulin signalling in the liver of 12-week-old male mice. Hepatic homogenates from mice injected i.p. with 0.75U insulin/kg (30 min) were analyzed by SDS-PAGE and immunoblotting using the indicated antibodies. Each lane on the SDS-PAGE gel represents an individual mouse.
(b-f) Organ weight to body weight ratios from 12-week-old male mice. Epididymal WAT (b), liver (c), kidney (d), pancreas (e) and heart (f). n=6-8 mice were used.
(g) Hypothalamic leptin signaling in 12-week-old female mice. Hypothalamic homogenates from mice injected i.p. with 2.5 mg leptin/kg (30 min) were analyzed by SDS-PAGE and immunoblotting using the indicated antibodies. Each lane on the SDS-PAGE gel represents an individual mouse.
(h) Whole-body weight variation upon ageing in female mice. n=6-8 mice were used. Grey square, WT; black circle, C2α$^{D1268A/WT}$.
(i-k) Perirenal WAT histology from 32-week-old mice. H&E of epididymal WAT sections are shown (i). Mean adipocyte areas (j) and adipocyte area distribution profiles (k) are also shown. Data are representative of perirenal WAT sections from individual mice. n=6 mice were used. Scale bar: 20μm. White bars, WT; black bars, C2α$^{D1268A/WT}$.
* $p < 0.05$; ** $p < 0.01$.
