# Supplementary material for: Inactivation of class II PI3K-C2α induces leptin resistance, age-dependent insulin resistance and obesity in male mice
Source: Diabetologia. 2016 Apr 30;59:1503–12. doi: 10.1007/s00125-016-3963-y (PMC4901096; doi:10.1007/s00125-016-3963-y)
Supplement: Supplementary file 3 — (PDF 267 kb) [file 125_2016_3963_MOESM3_ESM.pdf]

ESM Fig.2

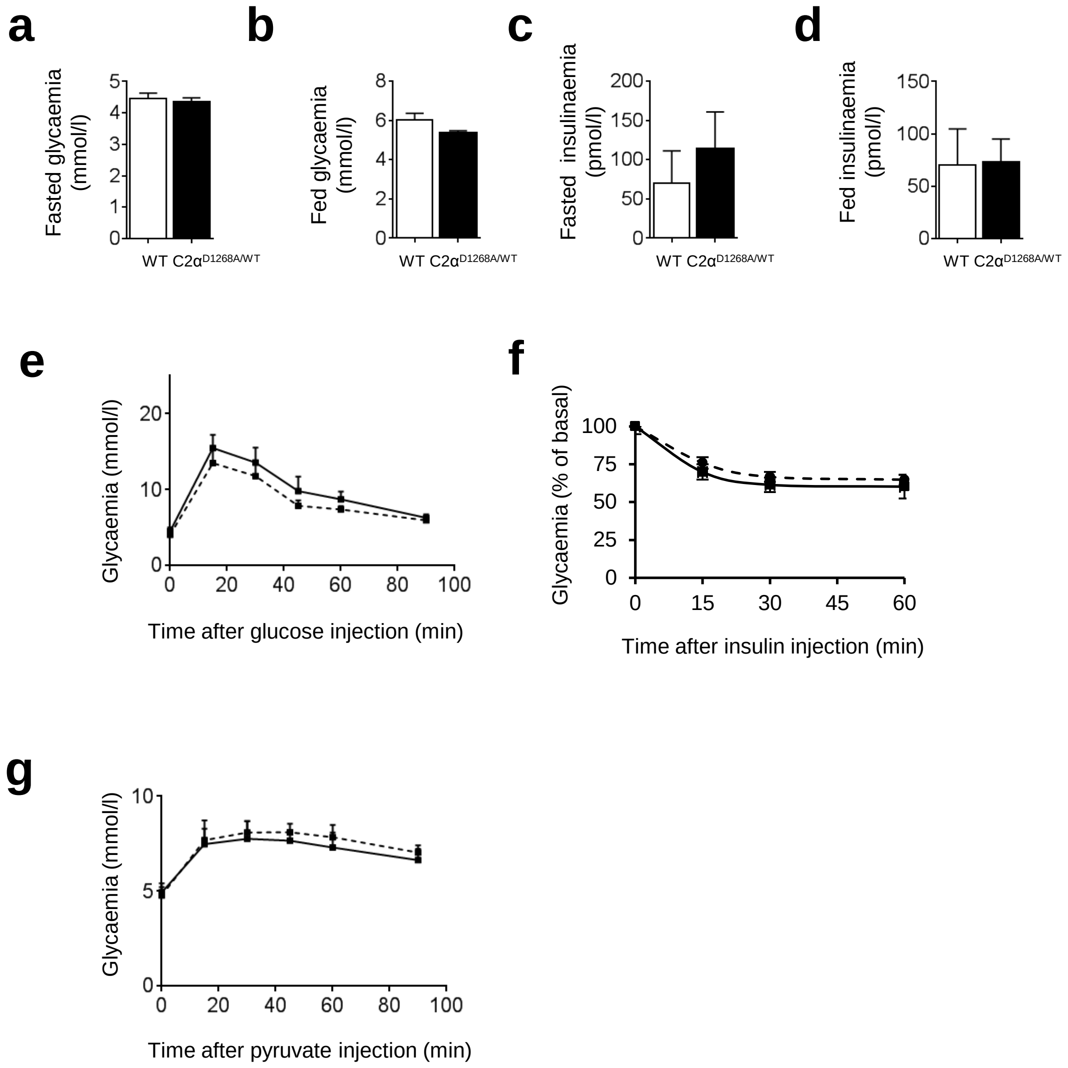
a
Fasted glycaemia (mmol/l)
WT C2α D1268A/WT
b
Fed glycaemia (mmol/l)
WT C2α D1268A/WT
c
Fasted insulinaemia (pmol/l)
WT C2α D1268A/WT
d
Fed insulinaemia (pmol/l)
WT C2α D1268A/WT
e
Glycaemia (mmol/l)
Time after glucose injection (min)
f
Glycaemia (% of basal)
Time after insulin injection (min)
g
Glycaemia (mmol/l)
Time after pyruvate injection (min)

Inactivation of class II PI3K-C2α induces leptin resistance, age-dependent insulin resistance and obesity in male mice

Electronic Supplementary Material

ESM Fig.2 Metabolic characteristics in WT and C2α$^{D1268A/WT}$ mice.
Data represent mean ± SEM.
(a-b) Glycaemia under fasted (a) and fed (b) conditions of 32-week-old female mice. n=12-23 mice were used.
(c-d) Plasma insulin levels under fasted (c) and fed (d) conditions of 32-week-old female mice. n=4-10 mice were used.
(e) GTT in 32-week-old female mice. Results shown are pooled data from 3 independent experiments using mice from different litters. n=9-13 mice were used.
(f) ITT in 32-week-old female mice. Results shown are pooled data from 2 independent experiments using mice from different litters. n=8-11 mice were used.
(g) PTT in 32-week-old male mice. n=6-9 mice were used.
Solid line,WT; dashed line, C2α$^{D1268A/WT}$.
