# Supplementary material for: Inactivation of class II PI3K-C2α induces leptin resistance, age-dependent insulin resistance and obesity in male mice
Source: Diabetologia. 2016 Apr 30;59:1503–12. doi: 10.1007/s00125-016-3963-y (PMC4901096; doi:10.1007/s00125-016-3963-y)
Supplement: Supplementary file 4 — (PDF 221 kb) [file 125_2016_3963_MOESM4_ESM.pdf]

Inactivation of class II PI3K-C2α induces leptin resistance, age-dependent insulin resistance and obesity in male mice

ESM Table 1 List of organs and tissues from C2α$^{D1268A/WT}$ mice subjected to histological examination (H&E staining).

adrenal glands	pituitary
aorta	prostate
bones (femur/stifle joint)	salivary gland
brain	seminal vesicles
brown fat pad (scapular and peri-aortic)	skeletal muscle (gastrocnemius)
epididymides	skin and mammary glands
eyes	small intestine (duodenum, ileum, jejunum)
gall blader	spinal cord
heart	spleen
ileum, jejunum	sternum
kidneys	stomach
large bowel (caecum, colon, rectum)	submandibular lymph nodes
liver	testes-ovaries/oviducts
lung	thymus/thymic area
mesenteric lymph nodes	tongue
oesophagus	trachea
pancreas	urinary bladder
parathyroids and thyroids	uterus/vagina/cervix
peripheral nerve (sciatic)	white fat pad (perigenital and perirenal)
